# Oral health and *Candida* carriage in socioeconomically disadvantaged US pregnant women

**DOI:** 10.1186/s12884-019-2618-7

**Published:** 2019-12-05

**Authors:** Jin Xiao, Colleen Fogarty, Tong Tong Wu, Naemah Alkhers, Yan Zeng, Marie Thomas, Moustafa Youssef, Lin Wang, Lauren Cowen, Hossam Abdelsalam, Anna Nikitkova

**Affiliations:** 1 Eastman Institute for Oral Health, University of Rochester Medical Center, 625 Elmwood Ave, Rochester, USA; 2 Department of Family Medicine, University of Rochester Medical Center, Rochester, USA; 3 Department of Biostatistics and Computational Biology, University of Rochester Medical Center, Rochester, USA; 4 Department of Forensic Medicine, North Sichuan Medical College, Nanchong, China; 5 College of Stomatology, Peking University, Beijing, China

**Keywords:** Oral health, Pregnancy, Yeast infection, *Candida*, Dental caries

## Abstract

**Background:**

Despite the well-documented associations between poor maternal oral health and increased risk for adverse birth outcomes and dental caries in children after birth, prenatal oral health care is under-utilized, especially among the underserved population. In addition, oral *Candida* has recently been suggested as a potential culprit for children’s dental caries, with evident maternal contributions. Therefore, this study aimed to obtain epidemiological data on the oral health and oral *Candida* carriage in a cohort of underserved US pregnant women, and reveal factors associated with their oral *Candida* carriage.

**Methods:**

Demographic-medical-oral hygiene practice data were collected. Comprehensive oral examination was conducted. Caries status and plaque index were recorded. Oral samples (saliva, plaque and swab) were processed to identify *Candida* species and *Streptococcus mutans* by culturing-dependent and -independent methods. Multiple logistic regression analyses were used to identify factors associated with oral *Candida* carriage and caries severity.

**Results:**

Eighty-two socioeconomically disadvantaged women (48 pregnant and 34 non-pregnant) were enrolled. More pregnant women (79.1%) had > = 1 untreated decayed tooth when compared to their non-pregnant counterparts (47.1%) (*p* = 0.01). The average number of decayed teeth in pregnant and non-pregnant women was 3.9 and 3.1 (*p* > 0.05). Caries severity was positively associated with race (African American vs. white), plaque index and salivary *Candida albicans* level. *C. albicans* was the most predominant/abundant *Candida* strain, with cheek and tonsil as the most common colonized sites. The detection of *C. albicans* was 56%/56% in saliva and 40%/47% in plaque of the pregnant and non-pregnant groups, respectively. Study women’s oral *Candida* carriage is positively associated with hypertension [*p* = 0.03, odds ratio = 14.47(1.28, 163.51)], decayed teeth number [*p* = 0.04, odds ratio = 1.31 (1.01,1.69)] and salivary *S. mutans* level [*p* = 0.03, odds ratio = 4.80 (1.18–19.43)].

**Conclusions:**

Socioeconomically disadvantaged US women are in need of improved prenatal oral health, a large proportion of them have untreated decayed teeth and high carriage of oral *Candida*. Due to the observed significant association between the decayed teeth number and oral *Candida* carriage, providing oral health care during pregnancy (including limiting decayed teeth) will not only improve women’s oral health, but also present as a promising approach to reduce oral *Candida* carriage in women.

## Background

Oral health during pregnancy is vital to expectant mothers and their children. Poor maternal oral health is known to be associated with increased adverse birth outcomes, particularly preterm and low birth weight deliveries [1, 2]. A systematic review analyzed data from 22 studies and 17,053 participants, and revealed the risk of giving preterm birth among mothers with periodontitis was 1.61 times compared to those without periodontitis (*p* < 0.001); additionally, the risk of delivering low birth weight infants among mothers with periodontitis was 1.65 times as to those without periodontitis (*p* < 0.001) [3]. Furthermore, receiving prenatal periodontal treatment presents potentials to reduce adverse birth outcome. A recent study reanalyzed the data from a previous birth cohort, and suggested that periodontal treatment provided to mothers with mild to moderate periodontal disease before 21 weeks of gestation may prevent preterm births [4].

Besides the association between maternal periodontal diseases and adverse birth outcomes, a strong correlation was also found between maternal carriage of dental caries related microorganisms and an increased risk for dental caries in children [5]. Dental caries is a multifactorial disease with microbial, host genetics, diet, and socio-behavioral determinants [6–9]. Dental caries initiated from the virulent dental biofilms/plaque formed on tooth surfaces [10]. Within the dental biofilms/plaque, oral cariogenic bacteria metabolize dietary carbohydrate; produce acid and initiate demineralization of tooth enamel [11]. Although an enamel remineralization process takes place when the enamel is exposed to salivary calcium, phosphate and fluoride ions, however when the demineralization exceeds the remineralization process, dental caries occur [12]. Understanding the acquisition of cariogenic microbes are vital to the development of improved preventive strategies.

*Streptococcus mutans* and *Lactobacillus species* have traditionally been considered the prime microbial risk markers for dental caries [13–19]. Chaffee et al. found the high maternal carriage of both salivary *S. mutans* and *Lactobacilli* is associated with nearly doubled children’s caries incidence versus low *S. mutans* and *Lactobacilli* mothers (cumulative incidence ratio: 1.9; 95% confidence interval: 1.1, 3.8), after adjusting socio-demographics, feeding and care practices, and maternal dental status [5]. Besides these traditional culprits for dental caries, recent research on the role of *Candida* species in early childhood caries (ECC) and its synergistic interaction with *S. mutans,* has shed new light on potential fungus-focused approaches to early prediction and subsequent prevention of ECC. *Candida* species (especially *C. albicans*) have often been detected at higher levels in the oral cavity of children with ECC, compared to caries free children [19–27], and is positively correlated with caries severity [28]. In a recent meta-analysis, we showed that children with oral *C. albicans* presented with > 5 times greater odds of experiencing ECC than children without this yeast strain [29]. More important, we have shown that mothers of the children affected by ECC also have high *C. albicans* carriage (> 80% detection in both saliva and plaque samples) [28].

Maternal contribution to children’s *Candida* colonization was demonstrated in several studies. The vertical transmission rate from mother to newborns ranges from 14% typed by electrophoretic karyotyping and restriction endonuclease analysis of genomic DNA with pulsed-field gel electrophoresis, to 41% typed by DNA fingerprinting using a *C. albicans* strain-specific DNA probe [30, 31]. We further identified that more than 60% of mother and children with severe ECC aged between 2 to 5 years old shared identical oral *Candida* strains [28].

Even though the benefits of maintaining good maternal oral health during pregnancy are well demonstrated, many mothers-to-be do not receive timely prenatal oral care [32]. A national survey [33] recently released by a dental insurance company, Cigna, found that 43% of women have not had a dental checkup while 76% admitted to suffering from oral health problems (pain, gum bleeding and oral infection) during pregnancy. Our data from a US oral health institute urgent care clinic showed that instead of receiving routine prenatal oral health care, more than 10% of local socioeconomically disadvantaged expectant mothers sought dental emergency treatment during pregnancy; the provided procedures to expectant mothers ranged from relieving orofacial pain, treating severe periodontal inflammation, treating severe tooth decay and its related maxillofacial infection. Furthermore, prenatal dental care utilization was lower among black women [34], ethnic minorities [35] and women with socioeconomic disadvantages [36]. Thus, oral health represents an important often-neglected heath disparity during pregnancy [37, 38].

Understanding the oral health condition of expectant mothers, particularly the ones with socioeconomic disadvantages can help us to design appropriate preventive and treatment strategies to help mothers gain better oral health during pregnancy, and provide further benefits to their children. Additionally, since controlling infants’ oral *Candida* carriage has been raised as a promising strategy in ECC prevention, understanding the factors associated with mother’s *Candida* carriage is essential to prevention of oral *Candida* colonization in infants. Therefore, this study was conducted to 1) obtain epidemiological data on oral health and oral carriage of *Candida* species in a group of US pregnant women with socioeconomic disadvantages, in comparison to their non-pregnant counterparts; 2) examine the relationship between demographic, medical and oral health status, and oral *Candida* carriage in pregnant women.

## Methods

### Study population

The study protocols were approved by the University of Rochester Research Subject Review Board (RSRB00056870 and RSRB00067191). All participants were informed of the study objectives and protocols, and gave written consent prior to study activities. Pregnant women were sampled from patients visiting the University of Rochester Highland Family Medicine (HFM) or Eastman Institute for Oral Health (EIOH). Control subjects, non-pregnant women, was a convenient sample collected from patients visiting the University of Rochester Eastman Institute for Oral Health. Both clinics, HFM and EIOH, serve a large body of low-income patients, with a range of 40% African Americans, 40% White and 20% others. The status of holding New York state-support medical insurance was used as a filter for the selection of women with socioeconomic disadvantages. The age and socioeconomic status of the subjects were used to match between the pregnant and non-pregnant groups.

### Sample size

We calculated the sample size based on the estimation that 60% of pregnant women have untreated decayed teeth, compared to 21.8–30.3% of women in the general population who have untreated decayed teeth, reported by CDC [39]. The average of the reported proportions, 25%, was used for the non-pregnant women group. A z-test with unpooled variance at alpha = 0.05 gives us a sample size to achieve 80% power comprised of 28 women in each group, a total of 56 study subjects.

### Eligibility

For the pregnant women group, individual who met for the following inclusion criteria and exclusion criteria were enrolled. For the control group women who are not pregnant, all inclusion and exclusion criteria apply except the items relate to pregnancy. Inclusion criteria included:1) Female, equal or older than 18 years of age. 2) Pregnant with singleton fetus and in her 3rd trimester (time period extending from the 28th week of gestation until delivery). 3) Eligible for New York state-supported medical insurance, which is determined by income level (≤138% Federal Poverty Line).

Exclusion criteria included:1) Subjects who have decisional impairment deeming incapable of making an informed decision about her participation in the study. 2) Subjects who received oral and/or systemic antifungal therapy within 90 days of the baseline study visit. 3) Subjects who have severe systemic medical conditions (e.g., HIV infection) that make them prone to yeast infections.

### Data collection, examination and sample collection

Data on demographics were self-reported by the study subjects. Data on the medical background and medications were collected through self-reporting and confirmed by electronic medical records. See Additional file 1 for the demographic-medical background. The medical background included: 1) physician-diagnosed systemic diseases, such as hypertension, diabetes, asthma, anxiety, depression, kidney disease, liver disease, etc.; 2) medications that subjects were taking when enrolled in the study; 3) smoking status (Y/N). Data on oral hygiene practice that were collected through a survey form. A comprehensive oral examination (caries score, plaque index and oral candidiasis) was performed by one of three calibrated dentists in a dedicated examination room at the University of Rochester clinics, using standard dental examination equipment, materials and supplies, under portable lighting. Dental caries was scored using decayed, missing and filled teeth (MDFT) according to the codes proposed by WHO Oral health surveys – basic methods, 4th edition, 1997 [40]. Dental plaque was assessed using the plaque index as described by Löe [41]. Oral mucosa was evaluated using oral candidiasis clinical diagnosis criteria and defined as pseudomembranous/erythematous forms [42]. Inter- and intra-examiner agreement for the evaluated criteria was calculated by Kappa statistics, and exceeded 83% at the calibration.

The whole non-stimulated saliva samples were collected by spitting into a sterilized 50 ml centrifuge tube. Approximately 1 ml of saliva was collected for each subject. Supragingival plaque from the whole dentition (all surfaces of all teeth) was collected using a sterilized periodontal scaler [28]. The plaque samples were suspended in 1 ml of a 0.9% sodium chloride solution in a sterilized Eppendorf tube. To examine the mucosal *Candida* infection site, for pregnant women with positive detection of salivary or plaque *C. albicans*, additional mucosal swab samples were collected from cheek mucosa, labial mucosa, dorsal surface of tongue, hard palate, and tonsils using FlocqSwabs (Copan Diagnostics, CA, USA) at the next study visit.

Vaginal *C. albicans* test was prescribed for 33 pregnant women by their physicians within a window of 12 months before entering our study; available vaginal *C. albicans* data was obtained through the electronic medical records.

### Quantification and identification of *Candida* spp. and *S. mutans*

After the sample collection, the clinical samples (saliva/plaque/swab) were stored on ice and transferred to the lab located at the University of Rochester Center for Oral Biology within 2 h for laboratory testing. The saliva and plaque sample were gently vortexed and sonicated to break down the aggregation before plating. The sonication cycle was repeated three times, with 10 sec sonication and 30 sec rest on ice. BBL™ CHROMagar™ Candida (BD, Sparks, MD, USA) was used to isolate *C. albicans* by incubating at 37 °C for 48 h. This medium permits presumptive identification of several clinically important *Candida* species including *C. albicans, C. krusei, C. galabrata, C. dubliniensis,* etc.*,* based on the colony color and morphology [43]. CHROMagar™ Candida culturing medium has shown high *C. albicans* detection sensitivity (98.6%) and specificity (98.8%) [42]. *S. mutans* was isolated using Mitis Salivarius with Bacitracin selective medium by incubating at 37 °C for 48 h and identified by colony morphology [44]. Colonies of *Candida spp.* and *S. mutans* on each plate were counted and recorded as colony forming unit (CFU). Additionally, *C. albicans* and *S. mutans* were further identified using colony polymerase chain reaction method. The probes used for *C. albicans* were forward primer 5’CGATTCAGGGGAGGTAGTGAC3’ and reverse primer 5’GGTTCGCCATAAATGGCTACCAG 3′. *The* probes used for *S. mutans* were forward primer 5′ TCGCGAAAAAGATAAACAAACA 3′ and reverse primer 5′ GCCCCTTCACAGTTGGTTAG 3′ [45].

### Data analysis

During data analysis, women in the study were grouped based on their pregnancy status and oral *Candida* status. The characteristics of the two groups (pregnant vs. non-pregnant; oral *Candida* positive vs. oral *Candida* negative) were compared using t-test for continuous data and Chi-square or Fisher’s exact tests for categorical data. One-way ANOVA and Turkey test was used to compare the *C. albicans* carriage between swab samples taken from different mucosal sites. Mann-Whitney U Test was performed to compare the difference of decayed teeth number in relation to other binary variables including pregnancy, smoking, inhaler use for asthma, and ethnicity (Hispanic and non-Hispanic). Spearman’s rank was used to measure the correlation between variables (decayed teeth number, age, plaque index, salivary/plaque *C. albicans* and *S. mutans* carriage). Cohen’s kappa was calculated to test the agreement between oral and vaginal *C. albicans* detection. A cumulative logistic regression analysis was used to test variables associated with caries severity among study subjects. The caries severity was grouped into three levels: 0 (no decay), 1 (<=3 decayed teeth) and 2 (> 3 decayed teeth). Multiple logistic regression analyses were used to identify predictors of *Candida* carriage among study subjects. Estimated odds ratios (OR) and 95% confidence intervals (CIs) were calculated for the variables that were statistically significant. The variables in the regression analyses were selected in multiple steps. We first selected a list of variables from the dental literature which might be potentially correlated with the outcome variables. Then, for the covariates like age, race, tooth brushing frequency, even though they have high *p*-values and are insignificant in the model, we still retained them in the model. Other variables, especially clinical characteristics, such as diabetes, asthma, hypertension, were selected based on *p*-values. We used a cut-off of 0.2 for the inclusion of those variables. All statistical tests were two-sided with a significance level of 5%. SAS were used for all statistical analyses.

## Results

### Oral health and *Candida* carriage among pregnant and non-pregnant women

A total of 48 eligible pregnant women and 34 non-pregnant women were enrolled in this study. None of the expectant mothers reported having dental exams since their pregnancy. Characteristics of the study population are shown in Table 1. No statistical differences was detected between the pregnant and non-pregnant groups in terms of age, ethnicity, medical background, smoking status, tooth brushing habit, decayed teeth, decayed/missing/filled teeth, and salivary *C. albicans* carriage (*p* > 0.05). Pregnant women’s plaque index and salivary *S. mutans* carriage was higher than non-pregnant women’s (*p* < 0.05). Strikingly, 79.1% of pregnant women and 47.1% of non-pregnant women had at least 1 untreated decayed tooth (*p* = 0.01); the average number of decayed teeth (DT) of the study pregnant women and non-pregnant women are 3.9 ± 3.8 and 3.1 ± 4.8; no difference regarding the decayed teeth number was seen between pregnant and non-pregnant groups (*p* > 0.05). Moreover, the average number of DMFT of the study pregnant women and non-pregnant women are 6.9 ± 6.4 and 7.5 ± 4.5 (*p* = 0.09).

**Table 1:** Demographic, medical and oral condition characteristics of study subjects by pregnancy status

Categories		Non-Pregnant (*n* = 34)	Pregnant (*n* = 48)	*p*-value
Age (year)		31.8 ± 6.9	27.1 ± 5.1	0.34
Race	African American	24% (8)	52% (25)	< 0.001
	White	53% (18)	17% (8)	
	Asian	21% (7)	8% (4)	
	Others	3% (1)	23% (11)	
Ethnicity	Hispanic	18% (6)	10% (5)	0.34
	Non-Hispanic	82% (28)	93% (43)	
Use of antibiotics > 1 months in the past 6 months (Yes)		0% (0)	4% (2)	0.23
Diabetes (Yes)		0% (0)	8% (4)	0.08
Asthma (Yes)		9% (3)	8% (4)	0.94
Hypertension (Yes)		9% (3)	17% (8)	0.31
Anxiety and/or depression (Yes)		9% (3)	19% (9)	0.21
Smoking (Yes)		18% (6)	13% (6)	0.52
Tooth brushing	Twice/daily	82% (28)	63% (30)	0.11
	Once/daily	15% (5)	35% (17)	
	<one/daily	3% (1)	2% (1)	
Plaque index		1.5 ± 0.9	1.7 ± 0.6	0.002
Untreated decayed teeth percentage		47.1% (18)	79.1% (38)	0.01
Decayed teeth number (DT)		3.1 ± 4.8	3.9 ± 3.8	0.33
Decayed, missing, filled teeth number (DMFT)		6.9 ± 6.4	7.5 ± 4.5	0.09
Salivary *S. mutans* carriage (10^6^ CFU/ml)		2.1 ± 4.5	1.3 ± 2.1	0.12
Salivary *S. mutans* carriage	No carriage	9% (3)	0% (0)	0.01
	1–10^5^ CFU/ml	38% (13)	19% (9)	
	> 10^5^ CFU/ml	53% (18)	81% (39)	
Salivary *C. albicans* carriage (10^3^ CFU/ml)		1.3 ± 2.6	1.4 ± 5.5	0.66
Salivary *C. albicans* carriage	No carriage	44% (15)	44% (21)	0.63
	1–400 CFU/ml	24% (8)	33% (16)	
	> 400 CFU/ml	32% (11)	23% (11)	

**Fig. 1:**
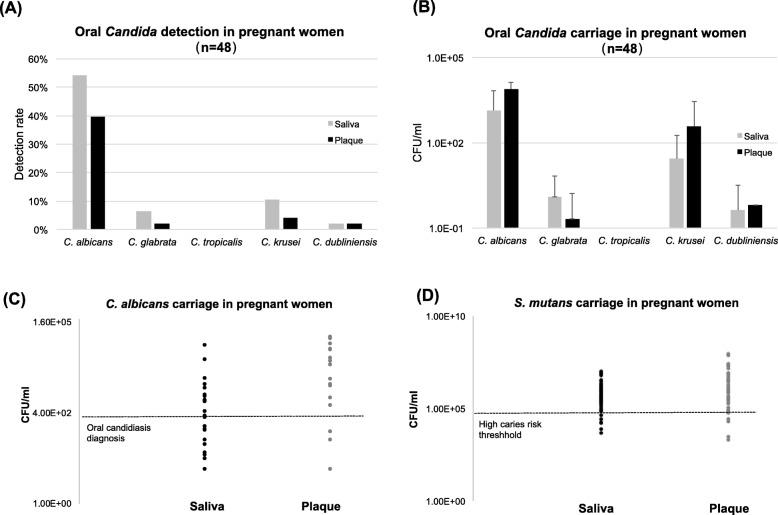
Oral *Candida* status in pregnant women; **a** Oral (saliva and plaque) *Candida* species detection in pregnant women. **b** *Candida* species carriage in the saliva and plaque of pregnant women. **c** Carriage plot of *C. albicans* in pregnant women with positive detection, a horizontal line indicating 400 CFU/ml is draw in the plot, which is an oral candidiasis criteria based on the salivary *candida* CFU established by *Epstein* et al [46]. **d** Carriage plot of *S. mutans* in pregnant women with positive detection, a horizontal line indicating 1.0 × 10^5^ CFU/ml is draw in the plot, which indicates a threshold of high risk for dental caries

Prevalence of oral *Candida* carriage is shown in Fig. 1 (pregnant women) and Additional file 2: Figure S1 (non-pregnant women). The overall *Candida* detection rates were similar: 56 and 56% in saliva, and 40 and 47% in plaque, in the pregnant and non-pregnant groups, respectively. *C. albicans* was the most predominant and abundant species found in *Candida* carriers of both groups (Fig. 1a, b, Additional file 2: Figure S1A and B). *C. glabrata*, *C. krusei*, *C. tropicalis* and *C. dunliniensis* were also detected among study subjects, with detection rates ranging from 3 to 11%.

Intriguingly, none of the study subjects were diagnosed as oral candidiasis upon clinical examination based on clinical diagnostic criteria, however when we plot the amount of salivary *C. albicans* carried in pregnant women group (Fig. 1c), we found more than 50% of the pregnant women could be diagnosed as oral candidiasis based on the salivary *Candida* CFU established by Epstein et al [46]. Moreover, 81% of pregnant subjects carried > 10^5^ CFU/ml of *S. mutans* in their saliva, setting them in the population who are at high risk for dental caries, shown in Fig. 1d.

### Oral mucosal site-specific *C. albicans* detection in pregnant women

**Fig. 2:**
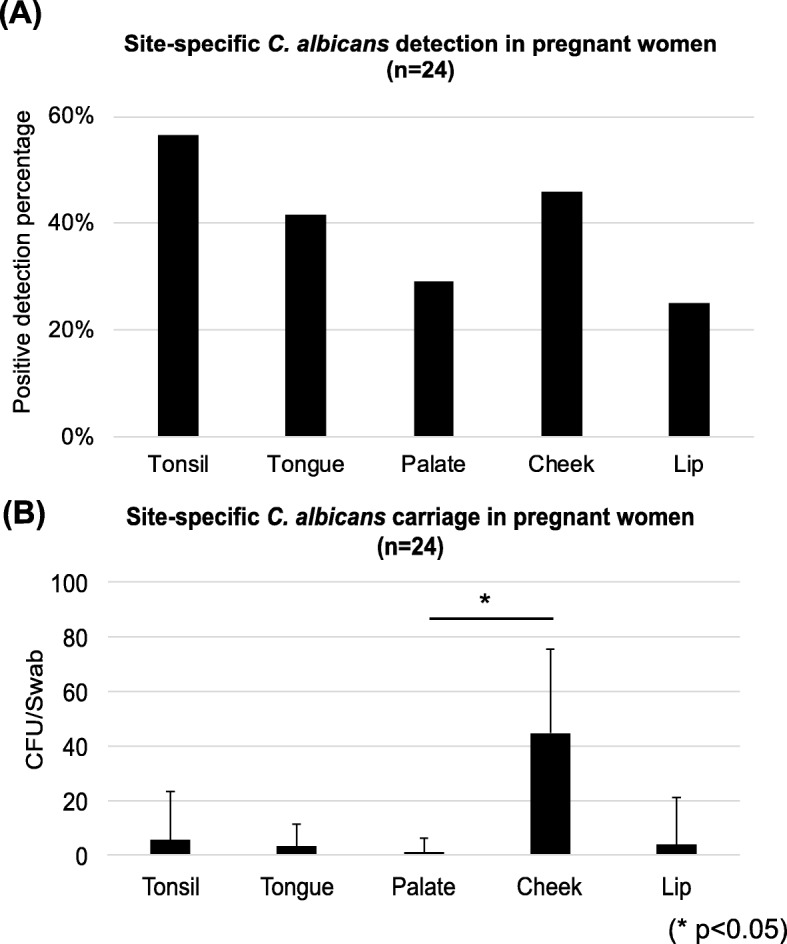
Oral *C. albicans* site-specific detection in pregnant women, The detection (**a**) and carriage (**b**) of *C. albicans* in different oral cavity sites of pregnant women were shown. Tonsil present as the most popular detection sites, followed by Cheek, tongue, hard palate and inner lip. Cheek has the most abundant carriage for *C. albicans*, with a statistically significant difference between the cheek and palate sites

Among 24 pregnant women who had positive *C. albicans* detection, we further determined the oral mucosal sites for *C. albicans* detection, results are seen in Fig. 2. Tonsil (57%) was the most prevalent site for *C. albicans* detection, followed by cheek (46%), tongue (42%), hard palate (29%) and inner side of the upper and lower lips (25%) (Fig. 2a). *C. albicans* was more abundant on the cheek mucosal surfaces when compared to the palatal mucosal surfaces, with a mean value of 13.21 ± 30.66 vs. 0.92 ± 5.24 CFU/swab (*p* < 0.05), shown in Fig. 2b. No quantitative differences were found between other mucosal sites (*p* > 0.05).

### Comparison between oral and vaginal *C. albicans* detection in pregnant women

**Fig. 3:**
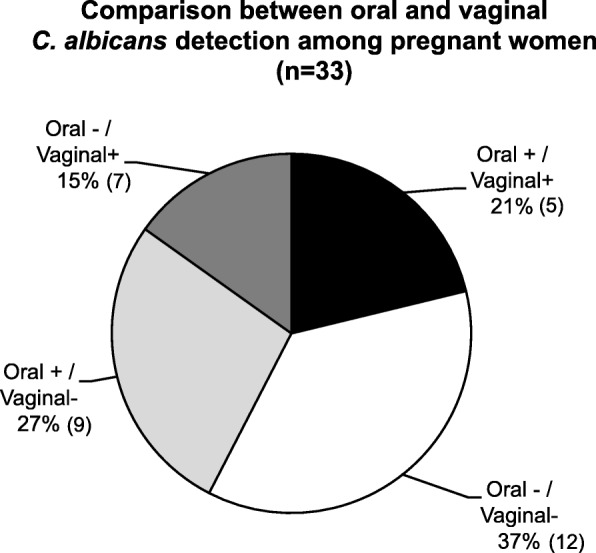
Comparison between oral and vaginal *C. albicans* detection among pregnant women, A comparison was made between oral and vaginal *C. albicans* detection among 33 study pregnant women. Nineteen (58%) pregnant women had consistent oral and vaginal *C. albicans* findings among which 7 (21%) pregnant women had positive *C. albicans* detection from both oral and vaginal sites and 12 (37%) had negative detection from both sites. Fourteen (42%) pregnant women have positive detection in only one site. Cohen’s kappa 0.144 (95% C.I. [−0.196, 0.485]) shows a slight agreement between oral and vaginal *C. albicans* detection

A comparison was made between oral and vaginal *C. albicans* detection among these 33 pregnant women (see Fig. 3). Specifically, 58% of pregnant women had consistent oral and vaginal *C. albicans* findings; 21% of the pregnant women had positive *C. albicans* detection from both oral and vaginal sites; 37% of pregnant women had negative *C. albicans* detection from both oral and vaginal sites. Whereas, 42% of the pregnant women had disagreement between the oral and vaginal *C. albicans* detection; 27% had positive oral *C. albicans* detection but absent for vaginal *C. albicans*; 15% had negative oral *C. albicans* detection but positive vaginal *C. albicans* detection. Cohen’s kappa 0.144 (95% C.I. [− 0.196, 0.485]) showed a slight agreement between oral and vaginal *C. albicans* detection.

### Factors associated with decayed teeth number in in women with socioeconomic disadvantages

The decayed teeth number significantly differed between these groups: ethnicity (*p* = 0.026) and inhaler use for asthma (*p* = 0.047); The decayed teeth number was marginally different between pregnant and non-pregnant groups (*p* = 0.051); no difference were detected between smoking and non-smoking groups (*p* = 0.27). The correlation between variables tested by Spearman’s rank is shown in Table 2. Decayed teeth number was significantly correlated with plaque index, salivary and plaque *S. mutans* level, and salivary and plaque *C. albicans* level. To analyze factors associated with caries severity in socioeconomically disadvantaged women, a cumulative logistic regression model was used. The factors input into the analysis were age, race, ethnicity, pregnancy, smoking, tooth brushing frequency, plaque index, salivary *Candida* level and salivary *S. mutans* level. Among all the factors, caries severity was positively associated with race (African American vs. White, *p* = 0.04, adjusted OR = 4.22, 95% CI = 1.07–16.57), plaque index (*p* = 0.001, adjusted OR = 4.37, 95% CI = 1.79–10.69) and salivary *C. albicans* carriage (*p* = 0.04, adjusted OR = 1.47, 95% CI = 1.02–2.11), detailed in Table 3.

**Table 2:** Spearman’s rank correlation between variables

	DT	Age	Plaque index	Salivary Ca CFU	Salivary Sm CFU	Plaque Ca CFU	Plaque Sm CFU
DT	1						
Age	−0.14	1					
Plaque index	0.44***	−0.07	1				
Salivary Ca CFU	0.38***	−0.02	0.15	1			
Salivary Sm CFU	0.40***	−0.08	0.32**	0.37**	1		
Plaque Ca CFU	0.35**	−0.04	0.14	0.075***	0.035**	1	
Plaque Sm CFU	0.36**	−0.11	0.44***	0.33**	0.72***	0.38***	1

*DT* Decayed teeth number

*Ca C. albicans*

*Sm S. mutans*

*CFU* Colony forming unit

**Correlation is significant at the 0.01 level (2-tailed)

***Correlation is significant at the < 0.001 level (2-tailed)

**Table 3:** Factors associated with caries severity in women with socioeconomic disadvantages

Variables		Estimate	Standard Error	Wald ChiSq	*p*-value	Odds Ratio	95% confidence interval	
							Lower	Upper
Age		0.003	0.04	0.003	0.95	1.00	0.92	1.10
Race	African American vs. White	1.44	0.70	4.25	0.04	4.21	1.07	16.57
	Asian vs. White	−1.03	0.94	1.22	0.27	0.36	0.06	2.23
	Other vs. White	0.15	0.90	0.03	0.87	1.16	0.20	6.82
Ethnicity (Non-Hispanic vs. Hispanic)		1.19	0.93	1.65	0.20	3.28	0.54	20.11
Pregnancy		0.57	0.63	0.82	0.37	1.77	0.51	6.15
Smoking		−0.70	0.84	0.69	0.41	0.50	0.10	2.60
Tooth brushing frequency		0.25	0.55	0.20	0.65	1.28	0.44	3.73
Plaque index		1.47	0.46	10.42	0.001	4.37	1.79	10.70
Salivary *Ca* CFU/1000 per ml		0.38	0.19	4.27	0.04	1.47	1.02	2.11
Salivary *S. mutans* CFU/100000 per ml		0.14	0.10	1.84	0.17	1.15	0.94	1.40

A cumulative logistic regression was used to test variables associated with caries severity in women with socioeconomic disadvantages. The caries severity was grouped into three levels: 0 (no decay), 1 (<=3 decayed teeth) and 2 (> 3 decayed teeth)

Ca *C. albicans*

Sm *S. mutans*

*CFU* Colony Forming Unit

### Factors associated with oral *C. albicans* carriage in women with socioeconomic disadvantages

To analyze the factors associated with oral *Candida* carriage in socioeconomically disadvantaged women, we further grouped all study women (both pregnant and non-pregnant) into two groups based on the *C. albicans* status - positive and negative groups. Characteristics of the study population in two groups are shown in Table 4. There were no differences (*p* > 0.05) between *C. albicans* positive and *C. albicans* negative group in terms of age, race, ethnicity, medical background, tooth brushing habit, and plaque index. More smokers were found in women with positive oral *C. albicans* detection (*p* = 0.04). Furthermore, higher percentage of having untreated decayed teeth, higher number of decayed teeth, higher number of decayed/missing/filled teeth, and higher carriage of salivary *S. mutans* were all found in the women with positive *C. albicans* detection (*p* < 0.05).

**Table 4:** Demographic, medical and oral condition characteristics of study subjects by *Candida* status

Categories		*C. albicans* positive (*n* = 46)	*C. albicans* negative (*n* = 36)	*p*-value
Age (year)		29.3 ± 5.8	28.8 ± 6.9	0.16
Race	African American	41%(19)	39%(14)	0.80
	White	35%(16)	27%(10)	
	Asian	11%(5)	17%(6)	
	Others	13%(6)	17%(6)	
Ethnicity	Hispanic	13%(6)	14%(5)	0.91
	Non-Hispanic	87%(40)	86%(31)	
Use of antibiotics > 1 months in the past 6 months (Yes)		0%(0)	6%(2)	0.11
Pregnancy (Yes)		56% (26)	61% (22)	0.68
Diabetes (Yes)		4%(2)	6%(2)	0.80
Asthma (Yes)		9%(4)	8%(3)	0.95
Hypertension (Yes)		20%(9)	6%(2)	0.07
Anxiety and/or depression (Yes)		20%(9)	8%(3)	0.15
Smoking (Yes)		22%(10)	6%(2)	0.04
Tooth brushing	Twice/daily	68%(31)	75%(27)	0.40
	Once/daily	28%(13)	25%(9)	
	< Once/daily	4%(2)	0%(0)	
Plaque index		1.7 ± 0.7	1.5 ± 0.7	0.89
Untreated decayed teeth percentage		80.0% (37)	53% (19)	0.008
Decayed teeth number		5.0 ± 4.8	1.8 ± 2.3	< 0.001
Decayed, missing, filled teeth number		8.5 ± 6.0	5.5 ± 3.7	0.016
Salivary *S. mutans* carriage (10^6^ CFU/ml)		2.1 ± 4.0	1.0 ± 2.1	0.046
Salivary *S. mutans* carriage	No carriage	0%(0)	11%(3)	0.02
	1–10^5^ CFU/ml	21%(8)	30%(8)	
	> 10^5^ CFU/ml	79(30)	59%(16)	
Salivary *C. albicans* carriage (10^3^ CFU/ml)		2.4 ± 5.8	0	NA
Salivary *C. albicans* carriage	No carriage	0%(0)	100%(36)	NA
	1–400 CFU/ml	52%(24)	0%(0)	
	> 400 CFU/ml	47%(22)	0%(0)	

Through multivariate logistic regression analysis (results shown in Table 5), the prevalence of oral *C. albicans* in women with socioeconomic disadvantages was significantly associated with hypertension condition (*p* = 0.03, adjusted OR = 14.47, 95% CI = 1.28–163.51), higher decayed teeth (*p* = 0.04, adjusted OR = 1.31, 95% CI = 1.01–1.69) and higher salivary *S. mutans* level (*p* = 0.03, adjusted OR = 4.80, 95% CI = 1.18–19.43). Other demographic, medical background, and oral hygiene practice characteristics were not associated with the prevalence of oral *C. albicans* in women with socioeconomic disadvantages, including pregnancy status.

**Table 5:** Factors associated with salivary *Candida* detection in women with socioeconomic disadvantages

Variables		Estimate	Standard Error	Wald ChiSq	*p*-value	Odds Ratio	95% confidence interval	
							Lower	Upper
Age		−0.03	0.06	0.25	0.61	0.97	0.87	1.09
Race	African American vs. others	0.48	1.04	0.21	0.64	1.62	0.21	12.38
	White vs. others	−0.31	1.04	0.08	0.77	0.74	1.00	5.62
	Asian vs. others	0.58	1.26	0.21	0.65	1.78	0.15	20.95
Ethnicity (Non-Hispanic vs. Hispanic)		−0.50	1.18	0.18	0.68	0.61	0.06	6.13
Diabetes		2.19	1.67	1.73	0.19	0.112	0.00	2.93
Asthma		0.68	1.07	0.40	0.53	1.97	0.24	16.13
Emotional disorder		0.94	1.00	0.88	0.35	2.56	0.36	18.12
Pregnancy		−1.18	0.82	2.07	0.15	0.31	0.06	1.54
Hypertension		2.67	1.24	4.67	0.03	14.47	1.28	163.51
Smoking		1.14	1.02	1.25	0.26	3.12	0.42	22.95
Tooth brushing frequency		0.66	0.72	0.84	0.36	1.93	0.47	7.87
Plaque index		−0.36	0.51	0.50	0.48	0.70	0.26	1.89
Decayed teeth		0.27	0.13	4.23	0.04	1.31	1.01	1.69
DMFT (Decayed, Missing, Filled teeth)		0.09	0.09	0.86	0.35	1.09	0.91	1.31
Salivary *S. mutans* level		1.57	0.71	4.82	0.03	4.80	1.18	19.43

Logistic regression model for was used to estimate the Maximum Likelihood and Odds Ratio of variables associated with the saliva *Candida* detection (yes or no). Hypertension, decayed teeth number and salivary *S. mutans* carriage level is significantly associated with oral *Candida* detection in women with socioeconomic disadvantages

## Discussion

Importantly, our study results indicate unmet oral health needs among US women with socioeconomic disadvantages, especially those who are pregnant. Upon examining the oral condition and *Candida* carriage among 82 US women with low socioeconomic status determined by their eligibility for state-support health insurance, a strikingly fact surfaced is that 79.1% of pregnant women had at least one untreated decayed tooth, with an average of 3.9 untreated decayed teeth per person. Based on the 2011–2014 CDC survey, 30.3% of women aged between 20 and 44 had untreated dental caries [39]. The caries rate in the study expectant mothers is significantly higher than the general US population. Untreated oral diseases among pregnant women with low socioeconomic status have been identified in the other states of the US as well. An interdisciplinary community-based oral health program in Florida US reviewed the dental encounter records of 180 underserved pregnant women in a Women, Infant, Children (WIC) program, and found 71.2% of the pregnant women had unmet dental care needs [47].

Second, we revealed a high oral *Candida* detection rate and carriage among the study women, 56% in pregnant and non-pregnant women, compared to a lower detection rate in healthy individuals (16–49%) [48, 49]. The average salivary *Candida* carriage was 1.4 × 10^3^ CFU/ml in pregnant women. Intriguingly, although none of the pregnant women presented clinical manifestation of oral candidiasis, more than 50% of them could be diagnosed as oral candidiasis based on the salivary *Candida* CFU established by Epstein et al [46], 400 CFU/ml in saliva. Our study results also indicate that with the tonsil being the most popular *Candida* detection site in the oral cavity, a combination of gauging and swishing oral antifungal rinse might provide better outcomes in treating oral candidiasis.

Third, upon examining the association between pregnancy and oral *Candida*, we found that pregnancy status is not significantly associated with *Candida* detection among the study population. This resonates with a previous study, in which pregnancy and diabetes independently did not influence the prevalence of fungi in the oral cavity and rectum of pregnant women [50]. While some efforts have been made to elucidate mechanisms by which pregnancy leads to changes in the composition of the oral microorganisms, the pathways remain unclear. The progesterone and estrogen have been suggested to affect the microbiota during pregnancy, but these effects have not been adequately demonstrated nor directly proven, other than the finding that estrogens enhance *Candida* infections [51, 52]. It is likely that the overall immune state during pregnancy plays a role leading to increased oral microbial load.

Besides the unclear mechanistic effect of pregnancy on the oral microbiota, the association between pregnancy and vaginal/oral microorganism carriage has revoked various discussions. For instance, one study revealed that oral microorganisms change during different stages of pregnancy. When comparing the abundance of seven common bacterial species in the oral cavity of non-pregnant women, early pregnancy, mid-pregnancy, and late pregnancy, the total viable microbial counts in all stages of pregnancy were higher than those of the non-pregnant women, especially in early pregnancy [51], and levels of the pathogenic bacteria *Porphyromonas gingivalis* and *Aggregatibacter actinomycetemcomitans* in the subgingival plaque, were significantly higher during the early and middle stages of pregnancy, compared to the non-pregnant group [51]. These results were further reinforced in an additional study, showing higher levels of *A. actinomycetemcomitans and Candida* in the second and third trimesters of pregnancy compared to non-pregnant women [53].

Fourth, we found that hypertension, decayed teeth number, and salivary *S. mutans* level are associated with oral *Candida* carriage in women, which have not been reported by previous studies. In recent years, with more research endeavors drawn to the association between oral *Candida* and tooth decay in children, research has indicated that children’s oral *Candida* carriage is associated with *S. mutans* level, and decayed teeth number. The association between *Candida* and dental caries identified in the children’s population might explain our findings in the adult population. This finding indeed intrigues an important clinical indication, which is restoring decayed teeth in mothers during pregnancy might offer therapeutic potential for controlling oral *Candida* in the mothers, preventing oral *Candida* colonization in infants by reducing the vertical transmission, and subsequently preventing dental caries in children.

Lastly, we examined the association between oral and vaginal *C. albicans* detection and indicated a high correlation, which is similar to some other study findings. *Lockhart* et al. [54] showed that among patients with recurrent vaginal candidiasis, 45% of them had identical oral and vulvovaginal isolates, 35% of them had related but not identical oral and vulvovaginal isolates, and 20% of them had unrelated oral and vulvovaginal isolates. The collective data from us and other studies indicated a cross-body sites contamination potential of *C. albicans*, between oral and vaginal habitats, which implies that the effective oral/vaginal *Candida* control strategy demands the consideration of both oral and vaginal origins.

The following limitations need to be considered when interpreting our study results: 1) With a cross-sectional study design, we only observed one time-point (the 3rd trimester) during the pregnancy and compared the oral condition and *Candida* carriage of the pregnant women to their non-pregnant counterparts. Although we observed that oral *Candida* detection is independent of the pregnancy, the results need to be confirmed by ideal study designs that examine all stages of pregnancy, including pre-, during, and post-pregnancy. 2) Limited sample size may have compromised the power of our multiple regression analyses. 3) The study was conducted in a single US city. Due to the nature of the single-site conducted study and small convenient sample size, the study results cannot be generalized to other populations. 4) The non-pregnant group was a convenience sample. The pregnant and non-pregnant subjects were enrolled from clinics where a majority of the patients are low-income individuals. The age and socioeconomic status were used to match the pregnant and non-pregnant groups. The status of holding New York state-support medical insurance was used as a filter for the selection of women with low socioeconomic status. The eligibility for state-support insurance is determined by income level (≤138% Federal Poverty Line). Although we did not conduct a case-by-case match for the education background, race, and ethnicity, there is no statistical difference in regard to the distribution of different races (*p* = 0.8) and ethnicity (*p* = 0.91) between the two groups (see Table 2). The current study focuses on the association, not causation, and we have controlled for the effects of covariates that might be potentially associated with the outcome variables in the models. We plan to match cases with controls using more variables and methods like propensity score in our future study.

## Conclusions

Socioeconomically disadvantaged US women are in need of improved prenatal oral health, a large proportion of them have untreated decayed teeth and high prevalence and carriage of oral *Candida*. Study women’s oral *Candida* carriage is positively associated with hypertension, decayed teeth number, and salivary *S. mutans* level. Due to the observed significant association between the decayed teeth number (caries severity) and oral *Candida* carriage, providing oral health care during pregnancy (including limit decayed teeth) will not only improve women’s oral health, but also present as a promising approach to reduce oral *Candida* carriage in women, and subsequently benefit their offspring.

## Supplementary information

**Additional file 1.** Demographic and medical background

**Additional file 2: Figure S1.** Oral *Candida* status in non-pregnant mothers. (A) Oral (saliva and plaque) *Candida* species detection in non-pregnant women. (B). *Candida* species carriage in the saliva and plaque of non-pregnant women.

## Data Availability

The datasets generated and/or analysed during the current study are not publicly available due to individual privacy but are available from the corresponding author on reasonable request.

## Abbreviations

CFU: Colony forming unit
CI: Confidence interval
ECC: Early childhood caries
EIOH: Eastman Institute for Oral Health
HFM: Highland Family Medicine
MDFT: Decayed, Missing and Filled Teeth number
OR: Odds ratio
