# Supplementary material for: Oral health and Candida carriage in socioeconomically disadvantaged US pregnant women
Source: BMC Pregnancy Childbirth. 2019 Dec 5;19:480. doi: 10.1186/s12884-019-2618-7 (PMC6896277; doi:10.1186/s12884-019-2618-7)
Supplement: Supplementary file 1 — Additional file 1. Demographic and medical background [file 12884_2019_2618_MOESM1_ESM.doc]

**Name** ________________________ **Birth date**________________ **Sex:** F M

**Race:** American Indian/Alaska Native Asian Native Hawaiian or Pacific Islander Black or African American

Caucasian More than one race unkown or unreported

**Ethinicity:** Hispanic Non-Hispanic

Home phone _____________ Cell phone _______________ Email address __________________________________________

Mailing address ____________________________________ City ____________________ State ________ Zip ___________

**Medical History**

**Does study subject has or had any of the following?
 (Please check any that apply)**

- Cancer or tumor
- Heart ailment or angina
- Heart murmur, mitral valve prolapse, heart defect
- Rheumatic fever or rheumatic heart disease
- Artificial joint or valve
- High or low blood pressure
- Pacemaker
- Tuberculosis or other lung problems
- Kidney disease
- Hepatitis or other liver disease
- Alcoholism
- Blood transfusion
- Diabetes
- Neurologic condition
- Epilepsy, seizures, or fainting spells
- Anxiety and/or depression
- Arthritis
- Herpes or cold sores
- AIDS or HIV positive
- Migraine headaches or frequent headaches
- Anemia or blood disorders
- Abnormal bleeding after extractions, surgery, or trauma
- Hayfever or sinus trouble
- Allergies or hives
- Asthma
- **NONE OF ABOVE**

**Allergies (Please check any that apply)**

- Latex materials
- Penicillin or other antibiotics
- Local anesthetics ("Novocain")
- Codeine or other narcotics
- Sulfa drugs
- Barbiturates, sedatives, or sleeping pills
- Aspirin
- Other:_____________________________________
- **NONE OF ABOVE**

**Medications (Please check any that apply)**

- Aspirin
- Anticoagulants (blood thinners)
- Antibiotics or sulfa drugs
- High blood pressure medicine
- Antidepressants or tranquilizers
- Insulin, Orinase,s or other diabetes drug
- Nitroglycerin
- Cortisone or other steroids
- Osteoporosis (bone density) medicine
- Other:_____________________________________
- **NONE OF ABOVE**

**Smoke or use chewing tobacco?**

❑ Yes

❑ No
