# Supplementary material for: Oral health and Candida carriage in socioeconomically disadvantaged US pregnant women
Source: BMC Pregnancy Childbirth. 2019 Dec 5;19:480. doi: 10.1186/s12884-019-2618-7 (PMC6896277; doi:10.1186/s12884-019-2618-7)
Supplement: Supplementary file 2 — Additional file 2: Figure S1. Oral Candida status in non-pregnant mothers. (A) Oral (saliva and plaque) Candida species detection in non-pregnant women. (B). Candida species carriage in the saliva and plaque of non-pregnant women. [file 12884_2019_2618_MOESM2_ESM.docx]

**Additional file 2: Figure S1. Oral *Candida* status in non-pregnant mothers**

(A) Oral (saliva and plaque) *Candida* species detection in non-pregnant women. (B). *Candida* species carriage in the saliva and plaque of non-pregnant women.
